# Recent Progress and Required Developments in Atmospheric Corrosion of Galvanised Steel and Zinc

**DOI:** 10.3390/ma10111288

**Published:** 2017-11-09

**Authors:** Ivan S. Cole

**Affiliations:** School of Engineering, RMIT University, Melbourne, Victoria 3001, Australia; ivan.cole@rmit.edu.au

**Keywords:** zinc, corrosion, oxides, droplets

## Abstract

This paper reviews the progress in atmospheric corrosion of zinc since 2009. It firstly summarises the state of the art in 2009, then outlines progress since 2009, and then looks at the significance of this progress and the areas the need more research. Within this framework, it looks at climate effects, oxide formation, oxide properties, pitting, laboratory duplication of atmospheric corrosion, and modelling. The major findings are that there have been major advances in the fields understanding of the structure of corrosion patina, in particular their layered structure and the presence of compact layers, local corrosion attacks have been found to be a significant process in atmospheric corrosion and experiments under droplets are leading to new understanding of the criticality of drop size in regulating atmospheric corrosion processes. Further research is indicating that zinc oxide within corrosion products may promote the oxygen reduction reaction (ORR) and that, in porous oxides, the ORR would control pore chemistry and may promote oxide densification. There is a strong need for more research to understand more deeply the formation and properties of these layered oxides as well as additional research to refine and quantify our emerging understanding of corrosion under droplets.

## 1. Introduction

There has been significant progress in our understanding of atmospheric corrosion of metals over the last decade. A range of studies into the interaction of aerosols with surfaces, oxide development, and the role of oxides in controlling the corrosion process have deepened the field’s understanding and together provide a fuller and more profound picture of atmospheric corrosion. To tackle this subject for all metals is too large a challenge for this modest paper so the developments will be highlighted by discussing the atmospheric corrosion of zinc. Wider developments will be incorporated when required to illustrate a common point. The current author was able to contribute a review on this subject in 2009 [1] and thus there is no need to repeat the material in that review, although it does form a useful definition of our knowledge a decade ago. In this paper for several critical processes that control atmospheric corrosion I will summarise the state of the art in 2009, highlight developments in the past decade, and discuss where greater research is required and what problems need to be resolved.

## 2. Climate Effects

### 2.1. State of the Art in 2009

Cole et al.’s [1] paper of 2009 extensively maps out aerosol formation, chemistry and transportation, and deposition while other papers [2] have outlined how an aerosol wets and other environmental effects on corrosion including solar radiation, rain, wind, etc. and how this is related to local and global climate so that, in 2009, a strong understanding of the effect of climate on corrosion existed. In this, it was appreciated that there was a variety of pollutant deposition mechanisms that could trigger corrosion, dry deposition, wet deposition through rain or fog, or deposition of aerosols.

### 2.2. Developments Since 2009

In two papers, Cole et al. [3,4] developed a corrosion map of Abu Dhabi and collected site climatic and corrosion data. Despite being very dry, significant corrosion was observed, higher than equivalent sites in Australia. The model indicated that this could be attributed in the low rainfall resulting in higher salt retention rates on the exposed metals combined and facilitating significant night time condensation. Chico et al. [5] have developed a corrosion map of zinc for Spain based on a dose function approach. Panchenko et al. [6] developed dose functions for predicting the corrosion of steel and corrosion for continental Russia based on data from 12 exposure sites. Tidblad et al. [7] looked at data for corrosion from 19 sights in Europe of a range of materials including steel, zinc, aluminium, and copper from 1987–2014. They found that the corrosion rates and SO_2_ levels significantly decreased after 1997.

Cole et al. [8] and Trivedi et al. [9] looked at the effect that climate change may have on metals in infrastructure in Australia. They used the effect of the latest climate scenarios on actual climate data to give predicted temporal climate sequences (rather than adjust average parameters). In general, they found that in coastal zones overall lower and less frequent rainfall would result in a higher level of retained salinity and thus a higher corrosion rate while in inland areas decreased RH would result in decreased night-time condensation and so decreased corrosion.

### 2.3. Significance of Developments and Need for More Research

The work up to 2009 and the recent work all indicated that the effect of climate on the atmospheric corrosion of zinc is best understood by studying how integrated temporal climate data affects local processes of corrosion. This is in contrast to the use of average climatic variables and parametric or regression methods. The processes controlling corrosion are highly dependent of cycles (wet/dry, etc.) which cannot be properly captured by average data and factor—such as wind, global radiation, and rain—all interact and so need to be studied by methods that facilitate this interaction.

## 3. Oxide Formation

### 3.1. State of the Art in 2009

It had been well established that the development of oxides can provide a barrier to reduce the corrosion rate of zinc or galvanised steel and that the effectiveness of this barrier depends on the nature of the exposure environment and of the oxide [10]. Most studies of oxides had looked at surface properties using such techniques as XRD, SEM-EDAX, or FTIR and thus a good knowledge of the nature of near surface oxides in short-term exposures and long-term field exposures had been obtained [11,12,13,14,15]. However, there were some minor differences in observation or theories of oxide formation based on the different nature of exposures. While a number of measurements had been made of oxide properties (surface wettability, IEP, electrochemical parameters) there was no consensus on which oxides were the most effective in reducing corrosion.

### 3.2. Developments Since 2009

A number of workers [16,17,18] have continued to undertake laboratory or field studies of oxide developing on the surface of zinc plates, though for some such as Odnevall et al. [16], the motive has widened to look at the quantity of zinc run off into the environment. Heldberg et al. [17] from Odnevall’s group studied zinc exposed for four years in marine and urban environments in Sweden. Using Raman spectroscopy, they observed that the corrosion products consisted mainly of hydrozincite and zinc oxide for both exposure conditions. However, while the ZnO was predominately crystalline in the marine location, it was predominately amorphous at the urban location. Cui et al. [18] examined the oxides developed when zinc was exposed for four years in tropical marine locations in China. They observed a three-layered structure with simonkolleite (Zn_5_ Cl_2_[OH]_8_·H_2_O) on the inner-most layer sodium zinc chlorohydroxy-sulfate (NaZn_4_Cl[OH]_6_SO_4_·6H_2_O) in the middle layer and zinc hydroxyl carbonates and zincite (ZnO) on the surface. They observed a decrease in corrosion rate with time which they associated with the protective properties of simonkolleite. Liu et al. [19] exposed zinc in a Chinese industrial marine site and noted that the corrosion rate decreased and the polarization resistance increased with time. The noted that, initially, the rust layer consisted of zinc hydroxyl carbonates while sodium zinc chlorohydroxy-sulfate and Zn_2_(OH)_15_ Cl_3_(SO4)_3_·5H_2_O compact layers developed with longer exposures. Interestingly, Liu et al. noted that the corrosion was initially localised but developed uniform coverage with time. Persson et al. [20] undertook a worldwide (Europe, East Asia, and USA) study of the corrosion of galvanised steel over periods from 0.5 to 2 years. Corrosion pits were observed for all exposure conditions and contained zinc hydroxyl-sulfates, sodium zinc chlorohydroxy-sulfate, and zinc hydroxyl chloride. Zinc hydroxyl carbonates were found outside the pits while, overall, the sulphate content was higher for industrial sites.

Li et al. [21] looked at salt deposition and corrosion for very short exposure times (30 min) in a marine Hawaiian locations on low carbon steel and zinc. In this, they duplicated the earlier test of Cole et al. [22] in Australia with very similar results. Li et al. [21] found that the corroded region or the original droplet was from 10–30 µm. Corrosion occurred at all sizes and secondary spreading was observed for the larger droplets. The most common corrosion product was simonkolleite with some zinc oxide and zinc hydroxyl carbonate with the latter two compounds being most common in the secondary spread zones.

One of the significant developments since 2009 has been the substantial use of focused ion beam SEM and TEM to study the full depth of oxides. Cole et al. [23] carried out one of the first studies of oxide development on zinc using FIB-SEM. They exposed zinc to fine seawater droplets under controlled humidity for 15 min to 6 h and observed the oxide that formed by Raman, XRD, SEM, and FIB-SEM. The SEM observations are consistent with their previous work but what was novel was their observation on the build-up of the oxide layer with time. There key observations were that:

(a) A three layer structure built up over the 6 h with consisting of a zone below the original metal surface where oxide has filled in previous areas of metal attack which appear to be along microstructural features, a zone above the original metal surface that is relatively compact, and a third zone above the first two where a highly porous crystalline structure is observed. The elemental composition of the first layer was predominately zinc and oxygen with some areas of zinc, oxygen, and chloride; the second layer contained zinc, oxygen, and carbon; and the third zinc, oxygen, carbon, and chloride.
(b) After the build-up of an initial oxide, the underlying metal is subject to localized attack that appears to be along grain boundaries.
(c) The oxide in the second and third layers appears to decrease in void content with time until it is relatively compact (except the upper surface) after 6 h.

Subsequently, Thomas et al. [24] in similar studies of zinc under a saline drop refined this layered structure. By both FIB-SEM and TEM analysis, he found a very fine (20 to 70 µm) crystalline compact layer (similar to that observed by McDonald [25], although this observation was after anodic polarization into the passive region in alkaline solution). TEM analysis indicated that the passive layer was ZnO. Above this compact layer was a precipitated layer of low void content and over this was a porous layer of zinc based chloride sulphate compounds. EIS studies indicated that the summation of these barrier layers was protective. In zinc substrates electrochemically oxidized in NaCl solution, Prestat et al. [26] observed similar compact layers to Thomas et al. The zinc was oxidized at a potential of −0.64 V in 0.1 M NaCl solution for 2 h. They observed a three-layered structure, the upper most layer consisted off flakes of simonkolleite (with pores between) underlayed by a compact layer of ZnO and an “interface layer” which was zinc-rich with some oxygen. Electrochemical studies indicated that the layer structure was partially protective. On the basis of their electrochemical measurements (cathodic polarization), Prestat et al. [26] postulate that the ZnO layer is inactive to cathodic dissolution and that only a fraction of the Zn sites are active for oxygen reduction.

In a recent paper, Thomas et al. [27] looked at the electrochemical behaviour and oxides that formed at high pH values. They conducted tests on zinc from pH = 12 to 13 in NaOH solution. They noticed a marked decrease in the anodic currents in potentio-dynamic scans and an even more marked decrease in corrosion current in potential hold tests as the pH was increased towards 13. FEB-SEM studies were undertaken of potential hold specimens and a parallel set of tests where droplets at pH = 12 were placed on zinc for times from 30 to 120 min. In all cases, compact oxides and precipitated oxides were seen. At short times, extensive voiding was apparent above or below the compact layer. For the droplet tests, the integrity of the compact layer was markedly higher after 120 min, voiding was substantially decreased, and the precipitation layer thickened.

Fattah-alhosseini and Mirshekari [28] also looked at zinc in alkaline solutions (0.01 M NaOH). Their study showed that Mott–Schottky analysis revealed that the passive films displayed n-type semi-conductive characteristics with the calculated donor density increasing with increases in the applied formation potential while EIS indicated that increasing this potential leads to the growth of a thicker but more defective film.

### 3.3. Significance of Developments and Need for More Research

Continued field studies are broadening or knowledge of the relation of locality and microclimate to oxide formation while the patterns observed prior to 2009 still holding in recent literature. Some finer details are emerging, such as observed climatic or geographic difference between where ZnO is found as a crystalline or amorphous phase. This could be quite important as crystalline ZnO can support the ORR (as discussed in a later section) and the barrier properties and defect transport mechanisms (particularly relevant to compact oxides) are likely to be different for the two phases.

The deepening understanding of the nature of zinc corrosion patina as a layered structures, where in some circumstances the bottom layer may be a compact oxide is critical. This indicates that surfaces methods of identifying corrosion product are not a reliable indicator of the full structure and may bear little relationship to the corrosion performance of this layered structure. A great deal more work is required in this area to generate a much greater understanding of how the layered structures form and how common and under what conditions the various elements form (particularly the compact oxide). Lastly, the role of each layer in forming a corrosion barrier needs to be elucidated.

## 4. Pitting

### 4.1. State of the Art in 2009

In 2009, it was generally held that zinc corrosion was a general process (rather than localised) although it was well established that oxide dissolution [15] was a critical element in on-going corrosion and that this could be local and that corrosion could initiate from local features [29] (e.g., grain boundaries or triple points) on the zinc surface.

### 4.2. Developments Since 2009

Recently, scanning vibration electrode technique (SVET) studies by Mena et al. [30] provided direct electrochemical evidence of pitting in zinc in laboratory tests. They used SVET to study the potential distribution on zinc in 1:1000 diluted ocean water. They observed localised anodic areas and more distributed cathodic areas after 2 h. Over the period of the test (5 h), the location of the anodic zones shifted. They attributed this to the development of oxides blocking the initial pit development and allowing the anodic (pitting) areas to redistribute to new locations.

Some workers have looked at pitting of zinc in specialized solutions, such as Abd El-Rehim et al. [31] in Na_2_S_2_O_3_ solution. These studies observed deep pitting and are primarily relevant to cases of high industrial pollutants.

Cole et al. [32] conducted an extensive study of zinc corrosion morphology and form across sites in Southeast Asia and Australia with severe marine, marine, severe industrial, industrial, marine/industrial, urban, and highland environments over a period of one year. Localized corrosion was observed at all sites and Cole et al. classified this localised corrosion in three modes depending on form and aspect ratio (α): very shallow α < 0.7, sharper pits α > 0.7 and nearly occluded pits. Interestingly, the results indicate that shallow pits occurred in the high SO_2_ environments and that sharp or occluded pits occurred in the moderate or low SO_2_ environments or marine locations. In the industrial environments where shallow pits occurred, S was found throughout the oxide, but where sharp pits occurred it was found almost exclusively at the bottom of the pits. Cole et al. observed strong oxide dissolution in industrial areas with high SO_2_ levels and they concluded that deposition of acid aerosols or rain locally dissolved the oxide, promoting relatively vigorous corrosion in these relatively large patches, leading to the formation of shallow pits. In contrast, in lower SO_2_ environments, the oxides remained intact and corrosion was facilitated by defects and voids in the oxide layer that lead to a highly localised Galvelle [33] type mechanisms and sharp or occluded pits.

### 4.3. Significance of Developments and Need for More Research

Recent research has demonstrated that localised pitting is very important to zinc corrosion. The question of whether this is to be regarded as ‘true pitting’ is somewhat of a definition issue as the aspect ratios of the local attack are much lower than the pits observed in stainless steel or aluminium. In field observations, pitting appears to be associated with local breakdown in oxide layers and thus the nature of oxide dissolution is critical to its form. Pitting-like phenomena have also been observed in the early stages of corrosion in laboratory experiments but this may have the added dimension of crystallographic orientation.

More work is required to assess how widespread such local corrosion is and what form it takes and how it is related to oxide breakdown and microstructural features. Importantly, the relationship between pitting-like and general corrosion in zinc needs to be elucidated.

## 5. Laboratory Duplication of Atmospheric Corrosion

### 5.1. State of the Art in 2009

In the 20th century, most laboratory studies of atmospheric corrosion where undertaken either in climate chambers or in solution. In the late 90s, it was realised that, it in order to closely duplicate actual field atmospheric corrosion, testing either under droplets or thin films was required.

### 5.2. Developments Since 2009

Valuable work has continued in studying corrosion of zinc under thin electrolyte layers (TELs) such as that of Cheng et al. [34]. In their study, they exposed zinc electrodes to 0.1 M NaCl with an initial thickness of 467 µm at RH of 75%, 85%, and 97% for 72 h. The solution will gradually evaporate, with evaporation being greatest at 75%. They found that the cathodic process was dominated by oxygen reduction and that initial corrosion rates were highest for the 75% RH exposure. However, this reversed with time and at 72 h the corrosion rates were highest for 97% RH exposure. The development of surface oxides was postulated to affect the corrosion rates.

Song et al. [35] used a three electrode system to investigate the electrochemical properties under a drop formed by the wetting of a NaCl crystal. The electrode system was a zinc working electrode (10 mm by 2 mm) and a reference and a counter electrode embedded in epoxy resin. This system was placed in a chamber at 97% RH, 25 °C for 2, 8, and 24 h. Polarization curves and EIS were undertaken, demonstrating that the corrosion rate decreased with time while the polarization resistance increased with time. EIS indicated that the system moved from one of charge transport control to one of mixed charge transport and mass transport control. The authors attributed this to the development of corrosion product which hindered mass transfer.

Azmat et al. [36] introduced a new technique to undertake high throughput conditions under droplets in order to better simulate corrosion under aerosols or rain. Multiple 0.5 µL (diameter approximately 1.6 mm) droplets of saline solution were deposited on a single zinc plate in a humidified environment for 6 h and then the volume loss (after etching) was measured with a profilometer. The observed mass loss (converted from volume loss) was very close to that calculated from multi-channel microelectrode analyser (MMA) measurement using same drop size and chemistry. They demonstrated the technique with MgCL_2_ and NaCl droplets of different concentrations (0.05 to 3.42 M), and acidified to a pH of 1 or not acidified. Interestingly, the 0.3 M MgCl_2_ acidified droplet showed the highest volume losses. In order to reproduce aerosol corrosion in a more accurate manner, Azmat et al. [37] inkjet printed very fine aerosol droplets with diameters ranging from 0.2 to 1 µm. Drop chemistry was seawater and NaCl solution at concentrations of 0.42, 0.6, and 3.42 M at its natural pH or acidified to a pH of 1 using a range of acids (HCl, H_2_SO_4_, HNO_3_, CF_3_SO_3_H) and held at 90% RH for 6 h. Corrosion rates at the lower salinities were relatively constant and not dramatically affected by acidification. With the 3.42 M solution, corrosion rates rose dramatically and varied considerable depending on acidified or not and the acidification method. Thus, the order of corrosion rates for the 3.42 M solutions were, in terms of acidifying agent: HCl, HNO_3_, unacidified, H_2_SO_4_, and CF_3_SO_3_H. Seawater demonstrated comparable but slightly lower corrosion rates with the order, again in terms of acidifying agent being: HNO_3_, H_2_SO_4_, natural, HCl, and CF_3_SO_3_H. SEM and FIB-SEM analysis of corrosion under the fine natural pH seawater droplets (1 µm) showed a porous oxide of 0.5 to 0.75 µm in thickness having elemental composition of Zn, O, and C. This compares with the more complex oxides containing Zn, O, C, Cl, S, and Mg that occur with larger (5 µm in diameter) droplets. Nazmat et al. attributed this difference to the high oxygen and thus high hydroxyl concentration likely throughout the small droplets that would promote significant CO_2_ absorption and precipitation of zinc hydroxyl carbonates. In larger drops, anode/cathode separation would occur and the rate of O_2_ diffusion would be lower and thus, at the anode, lower OH^−^ and carbonate concentrations would occur (these being higher at the cathode), promoting the precipitation of zinc hydroxychlorides and other more complex species.

Azmat et al. [38] undertook an extensive study of the nature of droplet, pH, stability, and corrosion product formation with droplets of moderate size (1 µL) that represent larger aerosols or raindrops. Droplets were placed on zinc held for 6 h in humidified conditions (RH = 90%). Droplet chemistry was 3.5% NaCl, 3.5%NaCl acidified to pH = 1 with either HCl or H_2_SO_4_, or alternatively natural seawater. Secondary spreading occurs for all droplets with the pH of the original drop showing significant spatial variation prior to secondary spreading. The edges of the drops all become less acidic or more alkaline while the central regions maintained or increased in acidity. Mass loss values were very different, being lowest for the sulphate-containing droplets (3.5% NaCl acidified with H_2_SO_4_ or seawater) and the highest for the unacidified NaCl solution or NaCl acidified with HCl. Within these two groups, the acidification increased corrosion rates only slightly. A combination of XRD, SEM, and Raman spectroscopy identified a wide range of corrosion products, with the main constituents being Zn_5_(OH)_8_(Cl)_2_·H_2_O (simonkolleite) in the central region of the non-sulphate containing solutions and NaZn_4_Cl(OH)_6_SO_4_·6H_2_O (gordiate) and simonkolleite being the major and minor components respectively in the sulphate containing solutions. ZnO, ZnCO_3_, or Zn_5_(CO_3_)_2_(OH)_6_ were the major constituents in the secondary spread region. The study has three notable results: that secondary spreading can occur even in acidic droplets, that there can be significant precipitation in such acidic droplets, and that corrosion rates under conditions that produce gordiate appear to be lower than those where the main product is simonkolleite. Risteen [39] refined the ink-jet printer method to deposit drops of a controlled size on low-carbon steels.

### 5.3. Significance of Developments and Need for More Research

This work has demonstrated that studies under thin electrolyte layers and droplets are useful and necessary for atmospheric corrosion and that standardized methods can be developed. The corrosion products formed under TELs or relatively large droplets appear similar to those observed in the field. Similarly, processes such as secondary spreading are observed for large droplets and moderate droplets both in laboratory experiments and in the field. However, when the droplets become small, corrosion products and reactions change. The exact size when this change occurs has not been determined, but it has certainly occurred by 1µm. For these droplets, the Evans effect with anode and cathode separation does not occur, nor is the ORR likely to be limited by oxygen diffusion. One result is the predominance of zinc oxide and zinc hydroxide as corrosion products.

Under many circumstances, atmospheric corrosion will just be the net effect of a large number of droplet surface interactions. Thus, it is important to determine the rate and forms of this type of corrosion. These studies are a start in this direction, but more work is required to define the corrosion rate as a function of drop size, the effect of enhanced oxygen levels (relative to corrosion in solution) on droplet corrosion, and the change and nature of the transition from Evans controlled to non-Evans processes. Importantly, droplet studies should be undertaken in cases where an oxide film has already built up—this is required to reproduce service effects as most exposed metals will have significant patinas.

## 6. Oxide Properties

### 6.1. State of the Art in 2009

There had been little direct study of the properties of oxides on zinc up until 2009. Most properties were inferred from electrochemical measurements of laboratory experiments or by combining mass loss data with surface determination of oxide properties. Because of the layered structure of the zinc patina, relating mass loss to nature of the surface oxide is unreliable. One exception to the lack of basic studies on oxides are the works of Muster et al. [40,41] who looked at surface charge, surface energy, and wettability on zinc corrosion products.

### 6.2. Developments Since 2009

Both theoretical and experimental researchers [42,43] have been concerned with how the development of crystalline ZnO may affect the oxygen reduction reaction. One school of thought arguing that, since ZnO is a semiconductor, it should support the ORR [44].

Thomas et al. [42] looked at this issue using a scanning electrochemical microscope (SECM) with a Pt microelectrode to measure the oxygen consumption in solution above a zinc sample. Here, a Pt tip is held at specific potentials where it undergoes the ORR to measure the local oxygen concentration. The experiments were conducted at pH of 7 in a 0.001M sodium carbonate salt solution and at pH of 13 in a 0.1 M NaOH solution. In the former condition, the zinc will be active electrochemically while in the latter it will be passive and covered by a zinc oxide film. At a pH of 7, the oxygen concentration measured at a distance of 50 µm from the zinc surface, using the Pt microelectrode, is 15–60% of the bulk oxygen concentration. Therefore, correspondingly, the oxygen consumption by zinc (due to ORR) is 40–85% of the bulk oxygen concentration. At a pH of 13, where zinc undergoes passivation, the oxygen consumption by zinc is 70–80% of the bulk oxygen concentration. Thus, the oxygen consumed and the ORR reaction in the passive state is considerably higher than in the active state. Thomas et al. argue that this is because significant oxygen reduction is occurring on the zinc oxide in the passive state. Nazarov et al. [45] looked at local corrosion of zinc and zinc alloys contaminated with NaCl crystals using a Kelvin probe. They found that the rate of ORR was high on oxide films that formed on zinc (which they attributed to the semiconducting properties of ZnO) but dramatically decreased if alloy agents such as magnesium were included in the zinc as these altered the composition of the resulting oxide films.

Prestat et al. [43] electrodeposited ZnO onto a copper and then investigated the ORR in 10^−3^ M KOH solution using a rotating disk electrode. They found that ZnO has a relatively poor electrocatalytic activity towards oxygen reduction and that oxygen reduction occurs via a direct pathway without a hydrogen peroxide intermediary. They also found that the ORR kinetics depend on film thickness and pH.

### 6.3. Significance of Developments and Need for More Research

The work on the role of zinc oxides in supporting the ORR reaction is vitally important for, as discussed in the next section, it has major implications the for the corrosion rate (when the corrosion rate is limited or controlled by the ORR) and the growth and densification of oxide, and thus their ability to develop barrier protection. In general, the properties of oxides on zinc are not well-defined and substantial work is required if our understanding of how the multi-layered structures occur is to be advanced.

## 7. Modelling

The aim of this section is not to look at the overall state of corrosion modelling but rather to consider any developments relative to zinc corrosion. The reader can refer to Gunnesagaran et al. [46] or Simillion et al. [47] for a recent detailed review of modelling.

### 7.1. State of the Art in 2009

A number of modelling schemes such as Graedel’s [48,49] GILDES or gas, interface, liquid, electrodic and surface regime approach or Cole et al’s [50] holistic model had been applied to zinc, while others such as Spence and Haynie [51] had modelled particular processes (oxide dissolution) and McDonald [25] had modelled void transport and oxide stability for passive films on zinc. These works provided a strong framework. However, how porous oxide and layered structures on zinc form and function had not been extensively studied.

### 7.2. Developments Since 2009

Venkatraman et al. [44] analysed electrochemical and diffusional processes occurring in a porous oxide. The model can thus provide guidance as to the processes that may occur as oxide layers build up and densify on zinc. They assumed that, in such a porous oxide, ORR could occur both at the bottom of pore at the metal surface and on the walls of the oxide surface (assuming the oxide was a semi-conductor such as zinc oxide). They analysed the percentage of the ORR that occurred in these two positions and found that these percentages were a function of oxide thickness, oxide conductivity, potential difference between the zinc surface and the oxide, and specific contact resistivity of the metal–oxide interface. Under appropriate circumstances, a significant-to-dominant fraction of the ORR can occur at the oxide pore surface. This in turn can change the chemistry in the pores, leading to depletion of oxygen in the pores and build-up of metal ions. The depletion of oxygen means that the ORR will move up the pore wall to the oxide solution interface. Although not proven by Venkatraman et al. [44], this change in pore chemistry could provide the conditions for precipitation or oxide growth in the pores, leading to the densification of oxides as highlighted by Cole et al. [23]. Sherwood et al. [52,53] used Kelvin’s approach to look at condensation and moisture retention in fine porous structures. They looked at under what conditions moisture would connect from the outer boundary of the oxide to the metal surface and defined a time of percolation which is the fraction of time (in a year) that that interconnected moisture exists. They found that, for certain oxide conditions, the time of percolation could be substantially higher than the time of wetness on an uncorroded surface.

Venkatraman et al. [54] modelled corrosion under films and derived the corrosion current density and corrosion potential as a function of kinetic, thermodynamic, and mass transport parameters. The model can be used in refining multi-scale models of atmospheric corrosion of zinc. Simillion et al. [55] developed a multi-ion transport and reaction model under thin NaCl containing films. Two important conclusions for this model were that the effect of macro-level geometric factors decreased as the thickness of the films decreased while chloride accumulation had a major role in controlling corrosion rates for thin films.

In very recent work of critical importance in understanding the resistance of compact oxides in zinc, Todorova et al. [56] undertook electronic structure calculations to look at the dominant defects that control the growth and dissolution of these oxide layers. Their calculations indicated that, in ZnO films, the doubly negatively charged oxygen vacancy may not be present and rather oxygen interstitial or unexpected charge states, such as the neutral oxygen vacancy, are found.

### 7.3. Significance of Developments and Need for More Research

These studies highlight some of the possible roles of porous oxides in the zinc system in terms of promoting longer wetting times, facilitating the ORR, and how the ORR on the pores of zinc oxide could promote densification of the oxide. All these works are preliminary and need further development. In particular, the porous oxide model of Venkatraman et al. needs to be expanded to include the tri-layer structures observed experimentally and in particular the compact oxide. It is notable that compact oxides are effectively grown in alkaline conditions in bulk electrochemical experiments yet form under droplet exposure in neutral conditions. It may be that the development of the porous oxide promotes sufficient alkalinity at the oxide–metal interface to promote the formation of a compact layer. Greater theoretical development is also required around how compact oxides function in zinc and how defects cross the oxide

## 8. Overall Significance of Developments and Need for More Research

This survey of the last decade’s literature on the atmospheric corrosion of zinc is by no means complete, but it is clear that there has been significant progress, particularly in:

(a) The development of corrosion patinas both in field and laboratory experiments and in particular the formation of multi-layered structures and compact layers.
(b) The identification of local attack as a significant phenomenon in zinc corrosion.
(c) Formulating methods to undertake corrosion under droplets and the critical role of drop size in determining the dominant corrosion processes and the oxides that form.
(d) Role of oxide layers, particularly ZnO, in promoting the ORR.
(e) The modelling or porous oxide and how the occurrence of ORR on oxide pore walls could have strong implications for pore chemistry and oxide densification.

Many of these elements are coming together and a new and more refined understanding of how atmospheric corrosion of zinc occurs is emerging. For instance, the modelling of porous oxides and growth studies of compact oxides in alkaline conditions may if combined help explain how compact oxides may occur in corrosion under neutral droplets (the local pH at the zinc/oxide boundary may be made alkaline due to ORR on the oxide pore surface). However, this new synthesis is very incomplete and substantial work is required to understand how the layered corrosion patinas form and what their properties are, as well as to grasp a detailed understanding of corrosion under droplets and other processes that reflect the actual corrosion events in service. A combined modelling and experimental approach would lead to the most profound advance.
